# Supplementary figures and images for: Microplanning improves stakeholders’ perceived capacity and engagement to implement lymphatic filariasis mass drug administration
Source: PLoS Negl Trop Dis. 2025 Mar 31;19(3):e0012105. doi: 10.1371/journal.pntd.0012105 (PMC11990631; doi:10.1371/journal.pntd.0012105)

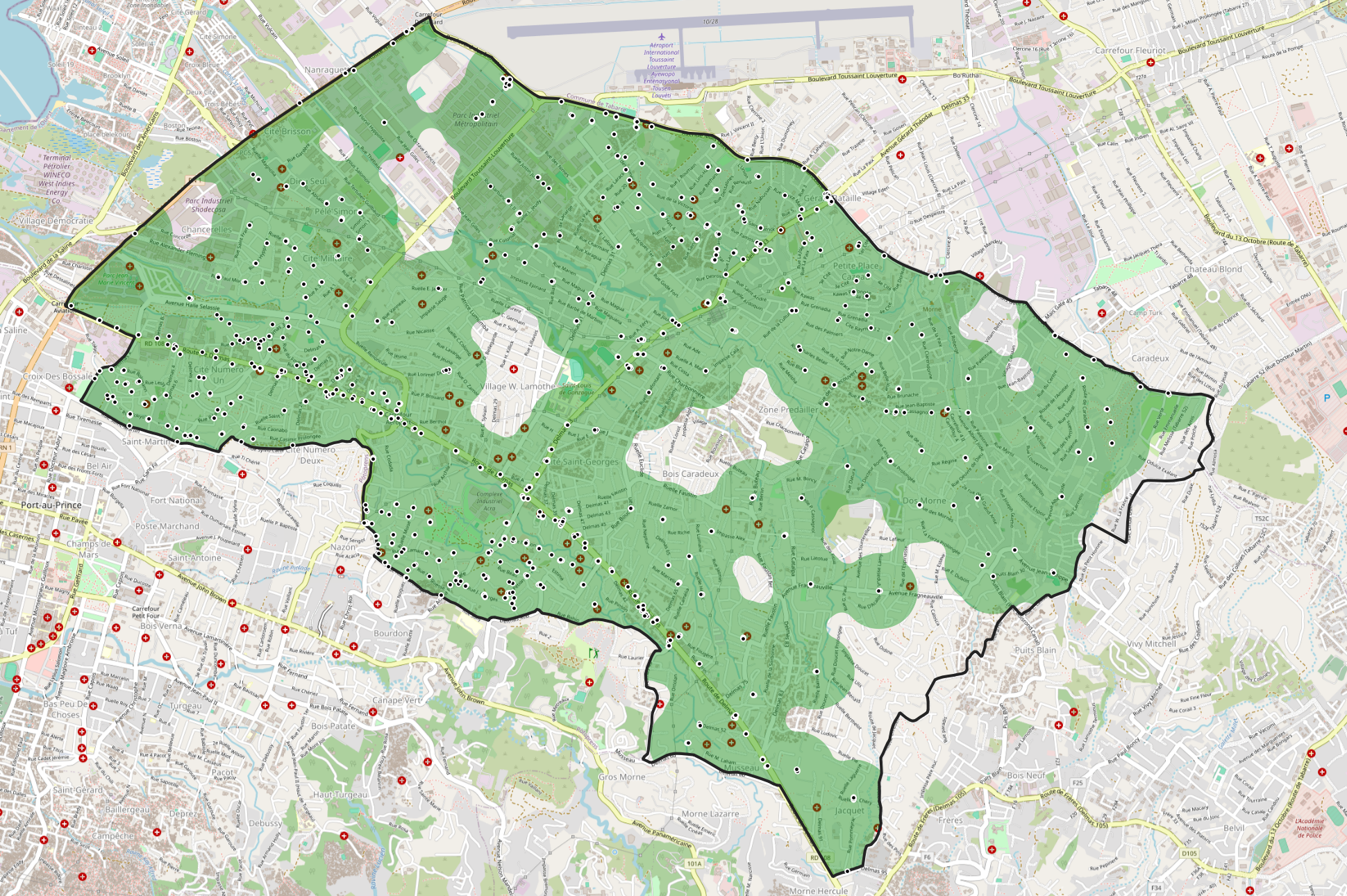

Supplement: S1 Fig — Analysis of access of the population to distribution posts during the 2017 mass drug administration (MDA) in Delmas commune in Port-au-Prince, Haiti. An illustrative map generated by the microplanning team to estimate the accessibility of distribution posts for the Delmas population. Global position system (GPS) coordinates of the 2017 MDA distribution posts, represented as black dots, were mapped for the commune of Delmas. The microplanning team aimed for participants to walk no further than 500 meters to reach a distribution post, a measure of distribution post access. A 300-meter buffer (green) was placed around each distribution post, which corresponded to an approximate 500 meter walking distance (Manhattan distance). Areas within Delmas that were not covered by the post buffers were targeted for re-allocation during the 2018 MDA. This map was created using QGIS. The base layer is available for download at: https://www.openstreetmap.org/#map=9/18.808/-72.905, under the following terms and conditions: https://osmfoundation.org/wiki/Terms_of_Use (TIF) [file pntd.0012105.s001.tif]

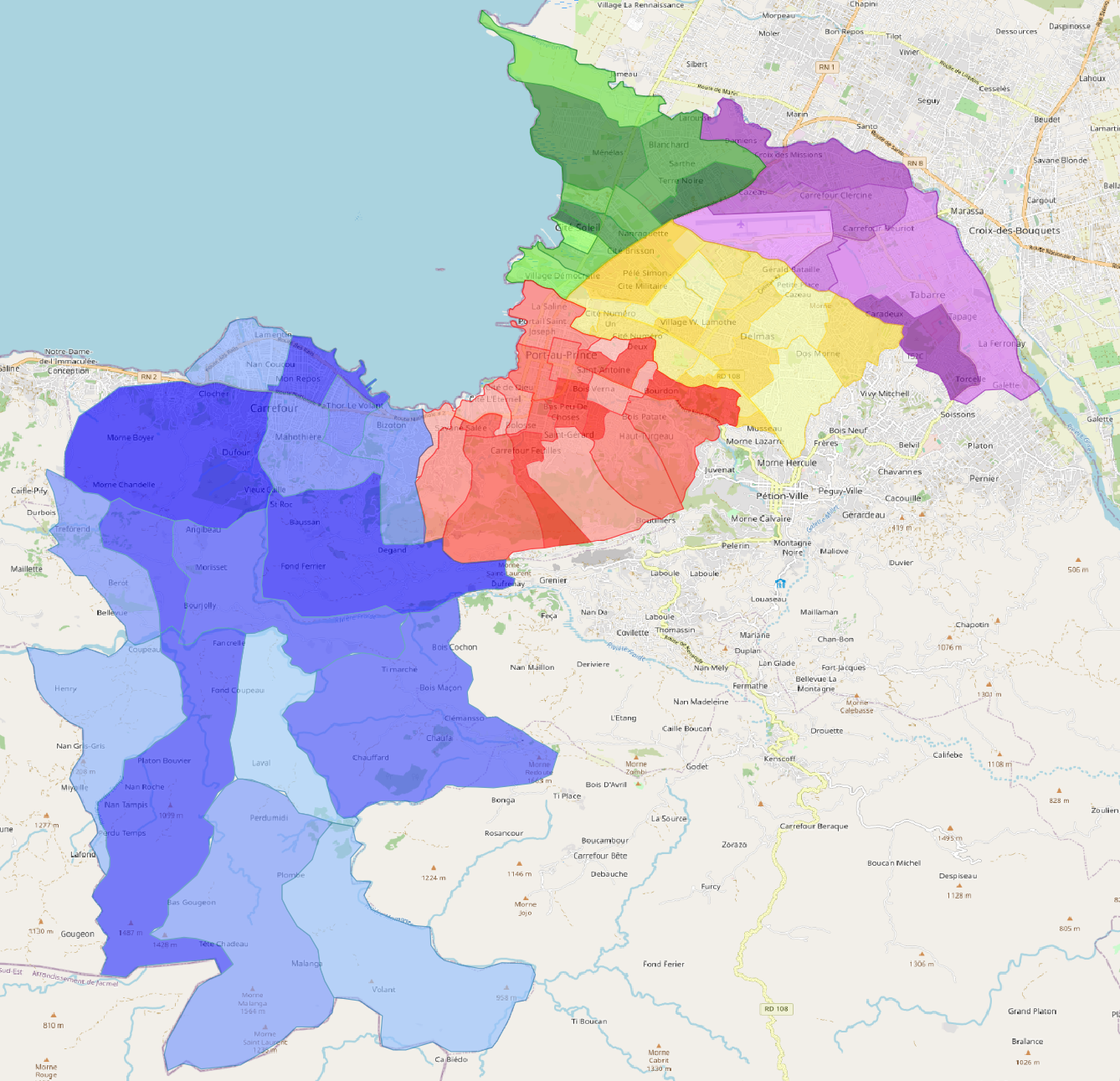

Supplement: S2 Fig — Final supervision area (SA) boundaries for 2018 mass drug administration (MDA) for community leaders (CLs) by commune in Port-au-Prince, Haiti. A map generated by the microplanning team in consultation with community leaders during microplanning workshops detailing SAs for each CL for five communes in Port-au-Prince. Areas are color coded by commune with shades of blue representing Carrefour, red representing Port-au-Prince, yellow representing Delmas, purple representing Tabarre, and green representing Cité-Soleil. This map was created using QGIS. The base layer is available for download at: https://www.openstreetmap.org/#map=9/18.808/-72.905, under the following terms and conditions: https://osmfoundation.org/wiki/Terms_of_Use (TIF) [file pntd.0012105.s002.tif]
