# Supplementary material for: Microplanning improves stakeholders’ perceived capacity and engagement to implement lymphatic filariasis mass drug administration
Source: PLoS Negl Trop Dis. 2025 Mar 31;19(3):e0012105. doi: 10.1371/journal.pntd.0012105 (PMC11990631; doi:10.1371/journal.pntd.0012105)
Supplement: Attachment — Submitted filename: PNTD-D-24-00455_ReviewResponse_20240923.pdf [file pntd.0012105.s007.pdf]

U.S. DEPARTMENT OF HEALTH AND HUMAN SERVICES

Public Health Service

Centers for Disease Control
and Prevention (CDC)
Atlanta GA 30333

Atlanta, September 23, 2024

Dear Drs. Ekpo and Clark:

Thank you for the careful review of our manuscript entitled "Microplanning improves stakeholders' perceived capacity and engagement to implement lymphatic filariasis mass drug administration" (your reference no. PNTD-D-24-00455). Below, please find our point-by-point replies to reviewer comments. Author replies are in bold with line references corresponding to the marked-up version of the revised manuscript.

Reviewer 1

Study area section- give a justification as to why the project was implemented in 5 of the 6 communes of Haiti and what criteria was used to select 5 and leave out one.

***Response*: Thank you for pointing this out. We agree that the statement was somewhat confusing as written. While six communes make-up the metropolitan Port-au-Prince, only five of the communes required mass drug administration (MDA) at the time of this project (i.e., Carrefour, Cité-Soleil, Delmas, Port-au-Prince, and Tabarre). The sixth commune, Petionville, passed the transmission assessment survey (TAS) in 2017 and is no longer required to conduct MDA activities. We have revised this statement to clarify this (see revised manuscript, lines 155-157).**

Overall design- please give more details the 10-2 days workshops held in phase 2- who moderated the workshops and who took the notes etc?

***Response*: Thank you for this suggestion. The workshops were moderated by the communal and departmental MSPP and implementing partner staff. The notes were recorded by implementing partner staff. We have added additional detail to include the personnel who moderated and took notes during the microplanning workshops (see revised manuscript, lines 179-186).**

Table 2 is titled as ‘demographic characteristics of the microplanning workshops participants” though it does not present any demographic characteristics such as age and gender but rather gives numbers and proportions of the participants from the 5 communes and their role. More details on gender for example would give some insights on the role played by gender in the microplanning workshops. Please give more details of socio-demographic characteristics. Was there an effort made to ensure that there was gender representation in the selection of the 356 stakeholders.

***Response*: Thank you for this comment. We agree that the heading of Table 2 did not fully correspond to the contents. Please note that the sex and age of the microplanning shop participants were not systematically collected, and hence, we cannot include those details in the manuscript. However, this is an important characteristic to collect in the future so that we may explore the role of gender in microplanning workshops. We will note this suggestion for future microplanning exercises. We have revised the heading of Table 2 to more appropriately reflect the contents.**

Discussion section from lines 348 to 392 requires literature review done so as to be able to compare with what the results of other similar studies show/report. In the introduction section the authors indicate that microplanning has been used in vaccination and immunization programs as well as HIV programs and such studies should be reviewed further to support the discussion section.

***Response*: Thank you for this suggestion. We have performed a more extensive literature review and contextualized our findings in light of results reported elsewhere, which predominantly derives from immunization programs.**

Each thematic area and sub-themes of the microplanning that led to important outcomes- collaboratively defining the supervision area, engaging stakeholders in identifying the MDA challenges, provision of previously unknown data and information and increase in community engagement should be discussed with a review of existing literature to support or contradict the findings being presented.

***Response*: Thank you for noting this shortcoming and suggestion to strengthen the discussion section. We have reviewed literature with a focus on the subthemes to better contextualize our findings.**

Three or so references/citations appear inadequate for the discussion section bearing the mind the various themes and sub-themes presented in the results.

***Response*: Thank you for noting the limited numbers of citations in this section. As suggested, we have performed a more extensive literature review and included those references throughout.**

Typos and grammar on lines 62, 171, and 352 (original lines).

On line 62, correct “that community that”
On line 171, correct “community- and school-based with distribution” statement is not clear
On line 352 correct “scare” resources with “scarce” resources.
***Response*: We thank the referee for reading our piece so carefully and noting these typo and grammatical issues. They have been addressed.**

Reference number 12 by Kisoka WJ et al., 2016 correct the typo “of mass drug administration” is repeated.

***Response*: Thank you for noting this duplication. It has been addressed.**

Check and adopt the accepted referencing style. Seems there are times when 3 or 4 authors of a journal have their names put before using et al., and times when only when the main author’s name is put and immediately followed by et al.

***Response*: Thank you for noting this inconsistency. We have reviewed author guidelines and now adhere to formatting and style of references in PLoS Neglected Tropical Diseases. To wit, we have ensured that the references follow the Vancouver style, e.g., listing all authors by last name and then up to two initials for their given names, rather than main author’s name followed by et al.**

Reviewer 2

A total of 356 stakeholders across 5 communities participated in the 2018 workshop, how many were involved in the previous work.

***Response*: The implementation of microplanning for MDA represented a pivot in the way that stakeholders were engaged in MDA planning. Through the microplanning exercise, stakeholders were engaged in a more formal and systematic manner, compared with past MDAs. As such, the number of stakeholders engaged in past MDA was not systematically documented.**

Method
“The objectives of the study clearly stated and include; to identify and address gaps in geographic coverage of supervision areas by reviewing the past MDA performance and proposes strategies to improve access to MDA.
The design of the study is appropriate. The sample size of the participants, stake holders and numbers of stakeholders meeting in the past MDA performance should be stated for a good comparison with the result of 2018 MDA performance.”

Response: **Thank you for this suggestion. We have included additional information to describe the characteristics of the 2017 and 2018 MDA (see revised manuscript, Table 4).**

"Lymphatic filariasis required an effective round of MDA with epidermiological coverage of at least 65% for a minimum of 5 rounds (5 years). The epidermiological coverage is determine by the number of individual ingesting the medicine at implementation unit (iu) population of people living in iu (eligible population targeted for MDA) to determine the gap in MDA coverage, there is need to state the targeted population of people living in the implementation unit and the number of individual ingesting the drug."

Response: **Thank you for this suggestion. We have added a table detailing the number of individuals ingesting the MDA medicines, the number of individuals in the target population, and the reported coverage by commune for 2017 and 2018, respectively (see revised manuscript, Table 4). It is worth noting that the implementing partner supporting the implementation of the Port-au-Prince MDA changed between the 2017 and 2018 MDAs. The number of individuals treated in 2017 is based on reports from the 2017 MDA partner. However, the 2017 MDA partner used a different data source to determine the targeted population figure than the source used to calculate 2018 MDA coverage. For the purposes of comparability between the 2017 and 2018 MDA, we re-calculated the 2017 MDA coverage results using the same data source that we used to calculate the 2018 coverage results. We note that these figures may differ from coverage figures reported elsewhere.**

"The statistical analysis method is appropriate but the post and pre-workshop differences should be tested with T-test at 95% confident level."

Response: **Thank you for your comment. Since our dependent variable was ordinal in nature, we used the nonparametric equivalent of the paired t-test (Wilcoxon signed rank test) to evaluate the statistical differences between the pre- and post-workshop responses. We have updated the methods to note that we tested this measure at the 95% level (see revised manuscript, line 232) and added this information to Table 3 to note that the p-value represents the Wilcoxon signed-rank test.**

"There is need for ethical clearance both in the community and school base stakeholders at least informed consent and permission should be sort from appropriate authorities."
Response: **This project has been evaluated by relevant authorities at Haiti's Ministry of Public Health and Population and the U.S. Centers for Disease Control and Prevention and determined to be a program evaluation. Please see the ethics statement for more details (see revised manuscript, lines 151-153).**

“The discussion are not strongly supported by other people works, all the same it is understandable that not much work has been done in the areas of microplanning. However, the limitation of the analysis should be clearly stated.”

***Response*: Thank you for this suggestion. As you point out, microplanning has only been implemented in limited contexts within the neglected tropical disease community. However, we have added additional citations from other contexts, such as immunization campaigns, into the discussion section.**

“Discussion from line 348-392- should be supported with citations and discuss properly References - Recheck references comply with the journal guideline especially with the use of " _et al_" and in formatting the reference list.”

***Response*: Thank you for this suggestion. We carefully reviewed the proposed style for PLoS Neglected Tropical Diseases and have ensured that the references follow the Vancouver style, i.e., listing all authors by last name and then up to two initials for their given names.**

“Recommendation: The paper should be considered for publication after the corrections.”

***Response*: Thank you for your favorable review of our manuscript.**

<u>Other comments embedded in the manuscript file:</u>

“Line 171 - Metion target population in the community and school, Examples, No. Of school”

***Response*: Thank you for this comment. We have added Table 4 to reflect the community populations for each commune. However, an accurate count of schools and their respective populations was not available to the authors. As part of the microplanning process, it was discovered that the list of schools maintained by the Ministry of National Education and Professional Training (MENFP) did not accurately represent the complete list of schools in the area, in particular, schools that were not registered with MENFP or satellite locations of registered schools were often not included on the school registers. One suggested action item from this microplanning exercise included conducting a census of schools to improve the understanding of the target population (Table 1). A census of schools in the target area was planned for 2019, however, it was unable to be carried out due to political insecurity.**

“Line 224 - 80.34 Gps were collected....Specify the number of participants”
***Response*: We have updated lines 237-238 to reflect this information.**

“Line 229 - 356 participants; Not same in day 1, Why??”

***Response*: All 356 participants were invited to participate in both Day 1 and Day 2 of the microplanning workshops. While all invitees participated in the workshops, only 339 (95.2%) submitted a completed microplanning evaluation that was analyzed for this report, which may account for discrepancies in the participant count.**

“Line 230- Specify target population”

***Response*: Thank you for this suggestion. We now provide an additional table that details the target population for all the communes who were targeted for this activity (see revised manuscript, Table 4).**

“Line 304, Table 3 - Authors can go further to test if the mean difference observed between pre and post workshop were significance at 95% confidence level, subjecting their mean/sd values to test.”

***Response*: Please see the response above for more details. The difference observed between pre- and post- workshop results were analyzed using Wilcoxen signed rank testing at 95% confidence level.**

“Recast: Line 383-348 & 401-403”

***Response*: We have revised the suggested sentences.**

We hope the revisions and replies above are satisfactory. Please do not hesitate to contact us if further discussion is needed. Thank you for your time; we look forward to finalizing this manuscript with you.

Kind regards,

CMWorrell

Caitlin Worrell

(Corresponding author, on behalf of all co-authors)
