# Supplementary material for: Microplanning improves stakeholders’ perceived capacity and engagement to implement lymphatic filariasis mass drug administration
Source: PLoS Negl Trop Dis. 2025 Mar 31;19(3):e0012105. doi: 10.1371/journal.pntd.0012105 (PMC11990631; doi:10.1371/journal.pntd.0012105)
Supplement: Attachment — Submitted filename: Microplanning improves stakeholders.docx [file pntd.0012105.s004.docx]

**Microplanning improves stakeholders’ perceived capacity and engagement to implement lymphatic filariasis mass drug administration**

The aim of the study was to improve stakeholder engagement in MDA microplanning so as to achieve adequate MDA uptake for Lymphatic Filariasis elimination in Haiti.The objectives were to 1. Identify and address gaps in geographic coverage, 2. Review past MDA performance and purpose to improve access, 3. Review roles and responsibilities of MDA personnel through stakeholder engagement. The study was done prior to the 2018 MDA and the results showed an improved MDA coverage and concluded that stakeholder engagement during MDA microplanning may improve personnel performance and contribute to achievement of MDA targets.

The authors report the results of a study on the role of stakeholder engagement in improving planning for increased MDA uptake. This is a very important component where stakeholders are engaged as part of the planning process for motivation and ownership of the MDA program. The paper is generally well presented.

**Comments**

**Study area section**- give a justification as to why the project was implemented in 5 of the 6 communes of Haiti and what criteria was used to select 5 and leave out one.

**Overall design**- please give more details the 10-2 days workshops held in phase 2- who moderated the workshops and who took the notes etc?

Table 2 is titled as ‘demographic characteristics of the microplanning workshops participants” though it does not present any demographic characteristics such as age and gender but rather gives numbers and proportions of the participants from the 5 communes and their role. More details on gender for example would give some insights on the role played by gender in the microplanning workshops. Please give more details of socio-demographic characteristics. Was there an effort made to ensure that there was gender representation in the selection of the 356 stakeholders.

Discussion section from lines 348 to 392 requires literature review done so as to be able to compare with what the results of other similar studies show/report. In the introduction section the authors indicate that microplanning has been used in vaccination and immunization programs as well as HIV programs and such studies should be reviewed further to support the discussion section.

Each thematic area and sub-themes of the microplanning that led to important outcomes- collaboratively defining the supervision area, engaging stakeholders in identifying the MDA challenges, provision of previously unknown data and information and increase in community engagement should be discussed with a review of existing literature to support or contradict the findings being presented.

Three or so references/citations appear inadequate for the discussion section bearing the mind the various themes and sub-themes presented in the results.

**Typos and grammar:**

On line 62, correct “that community that”

On line 171, correct “community- and school-based with distribution” statement is not clear

On line 352 correct “scare” resources with “scarce” resources.

Reference number 12 by Kisoka WJ et al., 2016 correct the typo “of mass drug administration” is repeated.

Check and adopt the accepted referencing style. Seems there are times when 3 or 4 authors of a journal have their names put before using et al., and times when only when the main author’s name is put and immediately followed by et al.
