# Supplementary material for: Microplanning improves stakeholders’ perceived capacity and engagement to implement lymphatic filariasis mass drug administration
Source: PLoS Negl Trop Dis. 2025 Mar 31;19(3):e0012105. doi: 10.1371/journal.pntd.0012105 (PMC11990631; doi:10.1371/journal.pntd.0012105)
Supplement: Attachment — Submitted filename: MicroplanningReviewerResponse_20250214.docx [file pntd.0012105.s008.docx]

Atlanta, 14 February 2025

Dear Dr. Clark:

Thank you for the careful review of our manuscript entitled “Microplanning improves stakeholders’ perceived capacity and engagement to implement lymphatic filariasis mass drug administration” (PNTD-D-24-00455). Please find below our point-by-point replies to Reviewer #3’s comments. Author replies are in bold with line references corresponding to the marked version of the manuscript.

Reviewer 3 – General Comments

This is an interesting programmatic use of microplanning for NTDs. There has been a surge of interest in examining ways to improve coverage for MDAs, and this adds a nice, practical example of what could be implemented. The paper is nicely written and generally clear; the majority of my comments were based on reading (and re-reading of certain paragraphs) that I could not quite understand as written.

***Response*: We thank Reviewer #3 for the positive appraisal of our piece. We hope that this manuscript will describe a practical experience using microplanning in the context of neglected tropical diseases (NTDs).**

That being said, there were a few areas that could use a bit more fleshing out: 1) from just reading the introduction, it was not clear that making maps/use of GIS was very central to the microplanning process; it was only once I got to the methods that I realized that. It would be helpful to bolster this part of the introduction;

***Response*: Thanks for pointing this out. As suggested, we have pulled this concept into its own paragraph and expanded on the topic (Lines 130-137).**

2) the authors indicate that they used a "retrospective pre-test" approach to minimize response shift bias, but couldn't social desirability bias have been introduced? If the literature shows otherwise, it would be helpful to discuss this;

***Response*: We felt like self-reported surveys were the only practical way to measure some of our microplanning aims, such as participants’ understanding of past performance and perceived engagement of microplanning participants by MSPP during the MDA planning process.**

**As Reviewer #3 notes, one limitation of self-reported surveys is the possibility of social desirability bias, which has been noted in the literature for retrospective pre-testing. We believe that traditional pre- and post-test may also be subject to social desirability bias, so we do not believe that this new methodology significantly exacerbates this issue.**

**We tried to mitigate the likelihood of bias, by using anonymous surveys with the intention that participants would be more likely to give honest feedback. As is noted below, some participants before-after pairs showed decreased scores which we believe indicates a willingness to provide honest feedback. We have added in the limitation section, that our results may be subject to social desirability bias (Lines 497-498).**

3) one of the measures used in the pre-post test was not clear "engagement by the MSPP in the MDA planning process." First, it's not clear to me what the definition used was, and second, whether it was really a fair question to ask/fair area for evaluation when most of the respondents were at the community level. This does require further clarification;

***Response*: Though both literature and previous experience in Haiti, we have observed that increased involvement and input into the planning and execution of MDA was motivating to MDA volunteers and ultimately improved the quality of the MDA itself. Hence, as part of this activity, we aimed to improve the two-way communication between the microplanning team and MDA volunteers and community members and to more diligently document and incorporate feedback and ideas. This process allowed the MDA strategies to be based on local expertise and experience that we hoped would better serve the underlying communities while at the same time being responsive to the needs of the MDA volunteers. We called this collective experience of eliciting their feedback and ideas, community engagement.**

**Despite the importance of this concept, we found it challenging to measure said engagement. We believed that self-reported surveys were the most feasible and practical way to capture this metric. We recognize that it has some limitations by being self-reported and not an objective metric.**

**We believe that it is a fair, though imperfect metric. Indeed, an estimated 88% of the microplanning participants had a direct role in the MDA implementation. Even those without a direct role, such as community members, provided valuable insight about the perspectives and preferences from their communities that were critical for the planning processes.**

**We have expanded on the concept to further clarify this for the reader (Lines 232-234).**

4) finally, in the discussion, the authors talk about digital tools being more cost-effective than traditional microplanning. This part of the discussion falls a little flat for me, given that the paper doesn't examine this at all (but expect the reader to feel that it would be more cost effective in this case as well), and in at least one of the citations given, cost-effectiveness seems to rely on use of DALYs--and I'm not certain that this would be equivalent for LF and vaccine-preventable diseases. But overall, an interesting paper and glad the authors have written this up.

***Response*: Thank you again for your positive review. While incorporating digital tools increased the cost of this exercise, we believe that this approach was worthwhile and may have increased the cost-effectiveness of our microplanning exercise. However, we recognize that the cost-effectiveness cannot be directly compared to between NTDs and vaccine preventable diseases.**

**Achieving high coverage is critical for both NTD and vaccine preventable diseases. Though we aim to achieve this while reducing the cost-per-dose delivered. Similar to vaccine preventable diseases, NTD programs aim to achieve minimum coverage targets and thus we see cost-per-dose-delivered as reasonably analogous between vaccines and NTDs. Please see additional information below.**

**Nevertheless, we have tempered the language (Lines 446-447).**

Reviewer 3 – Specific Comments

Abstract
Line 41: “ranked their attitudes…” Attitudes towards what?

***Response*: We have expanded on this point (Line 41).**

Lines 49-50: “…drug coverage increased…” Increased from 2017 MDA or all previous years of MDA?

***Response*: Thanks for pointing this out. We have clarified that this is compared to the MDA results in 2017 (Lines 49-50).**

Introduction
Lines 78-82 “Triple drug therapy…” Is IDA used in Haiti? If not, may not be a relevant statement. If it is, may wish to state that it is the strategy used in Haiti.
***Response*: Triple drug therapy has been adopted by the Ministry of Health as the preferred national MDA strategy within Haiti’s strategic plan for NTDs and the roll-out is still ongoing. We have revised this statement to make it clearer (Lines 78-79).**

Line 89: this is a really small point, but the phrase “…the majority of community member who did not receive the medication…” the “the” before medication is a little awkward, because readers of the present paper may not know what medication is being referred to if not involved in NTDs. Suggest simply removing the “the” before medication.
***Response*: We followed Reviewer #3’s suggestion and removed “the” (Line 91).**

Lines 89-91: In my reading of the paper cited, the most common reason for not being treated was absence at the time of the distribution. If this was related to “inopportune timing”, would suggest “…inopportune timing of the MDA which meant that many people were at their farms at the time of the MDA” (if I’m correctly recalling that paper).
***Response*: Reviewer #3 is correct that the MDA described in the citation was held during the harvest so many people were away at their farms during the distribution. This point has been clarified (Lines 93-94).**

Line 92: “insufficient time…” Insufficient time for CDDs to reach their distribution targets or cover their distribution areas?
***Response*: The respective sentence has been revised to enhance clarity (Line 96).**

Lines 110-113: “the create a realistic operational plan…as well as determine the material, financial, and human resources…” Most NTD programs conduct some sort of microplanning, though typically not in the way described in this paper. Instead, it’s typically a district compiling information provided by health center nurses, which are then rolled up to a district’s needs and then sometimes to regional needs. I’ve heard pushback from Program when discussing a more community-led or -involved approach that the budgets and resources they would ask for would be unrealistic. I wonder if the authors encountered this during this exercise at all, and if so, how it was managed?
***Response*: Reviewer #3 raises an interesting point. This project represented a shift in the strategy for planning MDA in Port-au-Prince, which previously looked like the process the referee describes. While we understand the concerns that some people may have about community-led planning processes, we believe there are strategies to balance community inputs and practical constraints. We believe that microplanning principles can be incorporated while recognizing that most MDA activities are constrained by externally set budgets, existing national policies, and other practical considerations. Within WHO’s microplanning manual, this accounts for the validation step and negotiation between the community and district/departmental/national level staff.**

**Overall, we found that the community was receptive to the participatory planning process, and we did not note major challenges in its implementation. During the process, microplanning participants were presented with some broad constraints and were asked to make suggestions within those constraints. While many of the participants’ suggestions were incorporated into the MDA plan, not every suggestion or request from community-led approach was adopted by the program, due to the budget constraints, scalability of the suggestion, or authority of the program to implement the change. For example, in Haiti, the number of distribution days was increased and the program added some teams to fill critical gaps in previously uncovered areas. The microplanning process mostly focused on optimizing the existing resources, such as ensuring an efficient distribution of distribution posts.**

Lines 119-120: “Neglected tropical disease programs…have applied microplanning less frequently…” I’m not certain NTD programs (or their partners) would entirely agree with this sentence as it reads now, as all of those I’ve worked with do some sort of microplanning prior to MDA. However, I would agree with this statement if referring to the community-level aspect/mapping aspects, as this often has not been part of developing these plans. Relatedly, is there a reference supporting the statement about financial constraints?
***Response*: Thank you for pointing this out. While we recognize that planning occurs to MDA almost universally, we have found that the systematic community-level planning process that was formalized and popularized by the immunization community is much less commonly implemented in NTD programs. We have found that different definitions and interpretations of microplanning is a challenge. We hypothesize that systematic microplanning is not routinely conducted in part due to financial constraints, however we do not have a specific reference to support that statement. Hence, we have removed the phrase “due to financial constraints” (Lines 123-124).**

Line 123: Given that the WHO NTD microplanning manual is meant to be disease agnostic, suggest removing “LF” and replace with “disease” or insert some language, such as “…or in the example of LF, assessments show that transmission remains despite multiple rounds of treatment.”
***Response*: We wholeheartedly agree that one of the values of this process is that it is disease agnostic. In the revised version this has been broadened, so that the statement covers MDA for all NTDs (Line 125).**

Lines 125-128: “Increasingly, digital tools…” Using digital tools really seems to be a central part of the microplanning process in Haiti and maybe why it was successful, but when I first read this section of the introduction, I wondered if these sentences even belonged, because they seemed to be tossed in for good measure. I wonder if pulling this into a separate paragraph and maybe talk specifically about the need to employ these tools in Haiti would be helpful and the help the reader to orient themselves to the fact that this was actual central to the microplanning process in the way Haiti conducted it.
***Response*: Indeed, digital tools were a critical component of the success of microplanning in the current study conducted in Haiti. Hence, we pulled this point into its own paragraph and expanded more on this topic in the introduction (Lines 130-137).**

Line 132: “initial rounds” can this be defined?
***Response*: We revised accordingly and provide a brief definition of “initial rounds” (Line 141).**

Line 138 “…the partners identified…” would it be better to state “the MSPP and its partners…”? otherwise, the language makes it sound as though the MSPP had no involvement in identifying strategies. In this case, the authors may be referring to “partners” as both the MSPP and implementing partners, but given that the term “implementing partners” was used in the previous sentence separately from the MSPP, it is not clear who was involved.
***Response*: Thanks for pointing out this critical omission. MSPP provided leadership and involvement throughout the microplanning process. We have revised the statement to clarify this (Line 147).**

Methods
Line 153: the font size used for the CDC human protection project identification number is different to the font used elsewhere.
***Response*: We thank Reviewer #3 for checking our piece so carefully. Font sizes have now been aligned throughout the manuscript.**

Line 162: “…is allocated to treat approximately 1,000 people…” allocated enough drug? Is asked to treat, 1,000 people?
***Response*: Teams are tasked with treating approximately 1,000 people, though for a variety of reasons, some teams treat more or fewer than that amount. A supply chain system is established to replenish medications and other supplies to ensure no stock-outs of medicines or critical supplies (Line 173).**

Lines 173-176: “we applied a 300 m buffer…” This sentence is not clear to me. Could this be shown in a figure?
***Response*: Thanks for the suggestion to create a figure to represent this concept (Supplemental Figure 1). In brief, we mapped a 300 m circular buffer around each of the distribution posts. This buffer represented 500 m of Manhattan distance, which we assumed to be a reasonable maximum walking distance for a participant. We used this graphic to identify areas where there were participants who were required to walk more than 500 m, which we considered an indicator of poor MDA distribution post access. We used this map to assist with re-allocating posts to minimize access gaps.**

Line 184: “voodoo temple” the other representatives listed are job titles/leadership roles, but “voodoo temple” is a place. Could it be “voodoo temple leaders” or “voodoo temple priests”? I’m not sure what the right title would be, but an actual position, job, or title seems to be needed here.
***Response*: Thanks for pointing this out. We have revised this to note that they were voodoo priests or *Ougans* (Line 198).**

Lines 186-191: in these few lines, the terms “MSPP staff,” “implementing partners,” “project staff,” and “program staff” are all used. IP and MSPP staff are clear—but not clear what is meant by “project staff” and “program staff.” May need to look at wording throughout so it’s clear what was the role of the MSPP and the role of the IP (and, if there are more categories, their roles).
***Response*: Thanks for pointing out this inconsistency. We have clarified that the team involved in the microplanning activity, consisted for national-level leadership from MSPP and implementing partner staff (Lines 179-182). We now use the term “microplanning team” throughout the paper for consistency.**

Lines 199-204: the text indicates that retrospective pre-testing reduces response shift bias. I have to admit, this is new terminology to me, but is there not a possibility that a social desirability bias could have been introduced, in which participants rate their new attitudes/understanding higher and their old understanding lower, so that the Program and Partners feel that the participants learned more than maybe they actually did?
***Response*: There are some advantages and disadvantages of using this strategy of evaluation. We aimed to reduce response-shift bias by using a retrospective pre-test strategy. Though the possibility of social desirability bias has been noted for the retrospective pre-test methodology. At the same time, we believe that any self-evaluation, including traditional pre- and post-test strategy, could be subject to social desirability bias. We added this in the limitations section (Lines 497-498).**

Lines 206-207: “engagement by MSPP in the MDA planning process.” It’s not clear what this measure means or exactly why this measure was used, and I’m wondering: is it a fair question to ask community-level workshop participants? Would they either really feel comfortable indicating low MSPP engagement or would they have a perception of what “MSPP engagement in the MDA planning process” entails?
***Response*: Since microplanning was one of several program strengthening activities during the 2018 MDA, we were hoping to isolate the success of this activity from the impact of the other initiatives. We went into the microplanning process with several aims. First, we were interested in ensuring several concrete elements and skills, such as understanding their zones of responsibility/influence and their roles and responsibilities in the MDA. Another important aim of microplanning was to better involve the community in the MDA planning process. Thus, we wanted to measure the extent to which the participants felt meaningfully involved, alongside MSPP, in the planning process. We felt like self-reported surveys were the only feasible way to measure this metric. However, self-reported surveys have several drawbacks, including social desirability bias.**

**All surveys were anonymous, which we think increased the likelihood that participants gave honest feedback. As was noted above, some participants before-after pairs showed a decrease in these metrics between the time points which hopefully indicates a willingness to provide honest feedback.**

Lines 263-264: “…including some modifications proposed by LF program staff…” This part of the sentence reads awkwardly. First, were LF program staff not instrumental in all modifications? Second, it introduces yet another term as per comment above re: “MSPP staff,” “project staff,” “program staff.”

***Response*: As noted above, we have harmonized the terminology throughout.**

Results -Figure 1
I appreciate that this figure was modified to ensure privacy, and description of the map indicates that it’s been lightly edited to anonymize the names of the CLs, but it seems the whole map is blurry. If it’s meant to be blurry to maintain anonymity, suggest making that explicit in the description.
***Response*: We validated all figure images through PACE during the manuscript submission process. However, we also notice that the image that is displayed in the document we downloaded from the review management software appears a bit blurry. However, when we click on the “download image” button a clear, full-resolution image is produced. We suspect that the document generated by the review manager produces a lower quality image for review purposes. We are prepared to work with the editors to ensure that all our images meet all quality requirements prior to publication.**

Figure 2
There doesn’t appear to be any discussion about respondents who ranked their knowledge/understanding of the 4 areas evaluated as lower in the second part of the retrospective pre-test (i.e., after the intervention). The numbers are fairly small, but it is curious that, for example, there were persons who stated their knowledge of supervisory areas was excellent prior to the microplanning process but poor afterwards. Could the authors include this in the discussion?

***Response*: We are also curious about the small number of respondents who indicated a reduction in the key metrics between the before and after time points. However, we do not have any additional information on the particular reasons that this small group of people reported reductions in scores.**

Table 1
2nd column, 4th row: “working conditions should be improved…” the decision/actions don’t really seem to be about improved working conditions but about having the CL and CP positions made official and therefore recognized by communities. “working conditions” to me sounds like labor law issues.

***Response*: Thanks for noting this area that needs clarification. We added additional information (Table 1: 2^nd^ column, 4^th^ row). With this element we tried to capture two separate but related issues. First, issues were raised in relation to working conditions. Second, participants noted a desire for increased compensation for volunteering for the MDA and also for environmental improvements such as an umbrella at each distribution post. Unfortunately, these were some of the requests that were unable to be met, which we noted in the discussion (Lines 320-321). The microplanning team explored with local companies the possibility of receiving a donation of umbrellas, but this was ultimately unsuccessful. We were able to make changes to improve the perception of the MDA volunteers among community members would help them convince individuals to participate in the MDA.**

2nd column, 10th row: “limited school information is available to staff…” which staff? MSPP staff? LF program staff? Other? Second, is the issue here that amounts of drug needed for schools can’t easily be quantified because the school populations are not known? Might want to make that explicit.

***Response*: We provide additional information to enhance clarity (Table 1, 2^nd^ column, 9^th^ row).**

2nd and 3rd columns column, 15th row “several areas had insufficient distribution post coverage” It’s not clear by the solution exact issue was—is it that more distribution posts are needed because of the mountainous areas? Is it that the distribution posts don’t maximize where people actually live?

***Response*: This involved both the addition of new posts and more efficiently allocating distribution posts to reduce coverage gaps. Additional information has been added (Table 1, 2^nd^ column, 9^th^ row).**

2nd and 3rd columns, 16th row: should “maximum” be “maximize”? and, will mop-up be implemented everywhere? If not was there a way suggested to prioritize mop-up areas?

***Response*: The respective part has been revised (Table 1, 2^nd^ column, 16^th^ row).**

Line 300: “several challenges…were unable to be addressed fully…” suggest brief reason for this is included and/or whether these issues will be reviewed at a later date.
***Response*: We have revised this section to include specific reasons why some suggestions were not adopted (Lines 320-322).**

Line 311: there appears to be an errant comma between “the” and “microplanning”. If you feel a comma is needed, suggest it is moved after “following.”
***Response*: We thank Reviewer #3 for checking our piece so carefully. The requested change has been made (Line 333).**

Table 4: The increase in coverage in Tabarre was quite remarkable and might warrant some further discussion. This was somewhat explained by Tabarre conducting its MDA first in the 2018 MDA—but was this the only reason? And, given that the reported coverage is well over 100%, does this either indicate denominator issues (and why would this only be a problem in Tabarre), or does it potentially indicate ongoing issues of supervisory areas?

***Response*: Tabarre was the first of the five communes treated during the MDA, followed sequentially by Cité-Soleil, Port-au-Prince, Carrefour, and Delmas. We noted that drug coverage was highest in Tabarre and decreased sequentially. While this may be an issue with the denominator, we believe that more likely it represented individuals captured in the numerator that were not represented in the denominator. Port-au-Prince is a large urban area with extensive daily population movement between communes as people move from their residences to places of employment or education. One of the major limitations of the post-based distribution strategy used in Port-au-Prince is that it is not possible to know if persons who were treated in Tabarre were residents of Tabarre commune. In an effort to maximize coverage, any eligible person who presented at a distribution post was offered treatment. Further, Tabarre has the smallest population, so even small numbers of neighboring persons treated have a larger impact on the coverage figure. We describe this limitation in the discussion (Lines 500-504). While it is a challenge to tease out this issue, we do believe that our results show a real increase in coverage since almost 600,000 additional people were treated across all five communes compared with 2017 coverage levels.**

Conclusions

Lines 402-403: “one such area…” is this sentence needed?
***Response*: This part of the sentence has been removed (Line 427).**

Lines 417-422: I think the authors are likely correct in their assessment of the importance of having the GIS to track coverage and reach targets. My question on cost-effectiveness, though, is: would the calculations be the same for NTDs as for vaccines? For example, in citation 35, part of the calculation for cost-effectiveness is in DALYs averted—childhood vaccines avert death or lifelong disability, in many cases, so the DALYs averted would be much higher than for morbidity that typically develops in adulthood. Additionally, since this paper is not showing the economics of microplanning for LF MDA, I might suggest tempering the argument about cost-effectiveness here.
***Response*: We believe that the cost-effectiveness is broadly applicable, but recognize that the cost-effectiveness cannot be directly compared to between NTDs and vaccine preventable diseases. In particular, calculating cost-per-DALY averted would be challenging and is likely lower for NTDs. The authors of the referenced paper weighed the increased costs of digital microplanning strategies against the changes in vaccine coverage achieved using digital microplanning methods. They then imputed cost-per-DALY averted using assumptions about the number of cases and associated death and disability that is assumed to be averted by increasing vaccine coverage.**

**Similar to vaccine coverage for vaccine preventable diseases, NTD programs use MDA coverage as one of the main process indicators. We see cost-per-dose-delivered as analogous between vaccines and NTDs. At the same time, calculating the DALY averted by an increased MDA coverage poses more of a challenge. That being said, since NTD programs are required to achieve minimum coverage levels for MDA rounds to be considered effective and to count towards the number of MDA rounds needed for stop-MDA surveys, we believe that efforts to increase coverage can also be cost effective if they ensure that each MDA round achieves the minimum coverage target. This can reduce the total number of rounds required before a district is eligible to proceed to a stopping-MDA survey.**

**We have tempered the language (Lines 446-447).**

Line 454: “…other areas looking to increase MDA coverage…” Do the authors mean other areas in Haiti, or other settings/other countries/other National Programs?
***Response*: We think these results are applicable to settings both across Haiti and in other countries. We have revised the text to reflect this (Line 479).**

Lines 458-460: “…this requires that sufficient time is available…” Can the authors outline the timeframe used in Haiti between the microplanning and the MDA to give other programs and partners an idea of how long this process took in Haiti?

***Response*: In our instance, microplanning took place approximately 3 months prior to the MDA. Microplanning took place from January – March 2018 (Line 167), while MDA took place between April and June 2018 (Line 391).**

We hope that our revisions and replies are satisfactory. Please do not hesitate to contact us if further discussion is needed. Thank you for your time; we look forward to finalizing this manuscript with you.

Kind regards,

Caitlin Worrell
